# Polymerase activity of hybrid ribonucleoprotein complexes generated from reassortment between 2009 pandemic H1N1 and seasonal H3N2 influenza A viruses

**DOI:** 10.1186/1743-422X-8-528

**Published:** 2011-12-12

**Authors:** Wai Y Lam, Karry LK Ngai, Paul KS Chan

**Affiliations:** 1 Department of Microbiology, The Chinese University of Hong Kong, Shatin, New Territories, Hong Kong Special Administration Region, People's Republic of China; 2 Stanley Ho Centre for Emerging Infectious Diseases, The Chinese University of Hong Kong, Shatin, New Territories, Hong Kong Special Administration Region, People's Republic of China

**Keywords:** Human swine influenza, Pandemic, Seasonal, PB1, PB2, PA, NP, RNP, RNA polymerase, Pathogenesis

## Abstract

**Background:**

A novel influenza virus (2009 pdmH1N1) was identified in early 2009 and progressed to a pandemic in mid-2009. This study compared the polymerase activity of recombinant viral ribonucleoprotein (vRNP) complexes derived from 2009 pdmH1N1 and the co-circulating seasonal H3N2, and their possible reassortants.

**Results:**

The 2009 pdmH1N1 vRNP showed a lower level of polymerase activity at 33°C compared to 37°C, a property remenisence of avian viruses. The 2009 pdmH1N1 vRNP was found to be more cold-sensitive than the WSN or H3N2 vRNP. Substituion of 2009 pdmH1N1 vRNP with H3N2-derived-subunits, and vice versa, still retained a substantial level of polymerase activity, which is probably compartable with survival. When the 2009 pdmH1N1 vRNP was substituted with H3N2 PA, a significant increase in activity was observed; whereas when H3N2 vRNP was substituted with 2009 pdmH1N1 PA, a significant decrease in activity occurred. Although, the polymerase basic protein 2 (PB2) of 2009 pdmH1N1 was originated from an avian virus, substitution of this subunit with H3N2 PB2 did not change its polymerase activity in human cells.

**Conclusions:**

In conclusion, our data suggest that hybrid vRNPs resulted from reassortment between 2009 pdmH1N1 and H3N2 viruses could still retain a substantial level of polymerase activity. Substituion of the subunit PA confers the most prominent effect on polymerase activity. Further studies to explore the determinants for polymerase activity of influenza viruses in associate with other factors that limit host specificity are warrant.

## Background

In April 2009, the Centers for Disease Control and Prevention (CDC) at Atlanta reported that a new influenza virus was found in Mexico and the United States [1]. The new influenza A H1N1 virus was soon characterized [2,3] to be a triple reassortant derived from human, avian and swine influenza viruses [3-5]. The virus spread rapidly worldwide [6] and the World Health Organization (WHO) declared that the pandemic has reached phase 6 on June 11 2009 [7]. Currently, the virus is still circulating worldwide [7].

Influenza viruses exhibit a restricted host range with limited replication in other species [8-10]. However, on rare occasions, influenza viruses can cross species barrier and adapt to a new host giving rise to a new lineage. Adaptation to a new species is believed to require multiple point mutations or reassortment of gene segments, or both. The molecular mechanism and genetic determinants that restrict, or permit, the replication of influenza viruses in humans remain unclear. While host haemagglutinin receptor specificity is clearly an important factor, it is not an absolute barrier to cross-species infection [11-13]. Growing evidence suggests that viral polymerase and nucleoprotein (NP) play a pivotal role in determining host selection and adaptation [13,14].

Replication and transcription of influenza RNA segments are regulated by a virus-encoded RNA-dependent RNA polymerase [14]. The polymerase is a heterotrimeric, multifunctional complex composed of three viral proteins, polymerase basic protein 1 (PB1), polymerase basic protein 2 (PB2), polymerase acidic protein (PA), which together with the viral NP form the viral ribonucleoprotein (vRNP) complex that is required for viral mRNA synthesis and replication [14]. PA is an endonuclease [15-19], and involves in promoter and cap binding [20,21]. PB1 contains active sites for nucleotide elongation [22,23] and binding to promoters of vRNA and cRNA [22,24,25]. PB2 involves in cap-snatching from host mRNA [26,27], and has been the focus of host adaptation and pathogenicity study. PB2 mutation, particularly the E627K, has been linked to the adaption of avian viruses to mammalian host [28,29]. Another PB2 mutation, D701N, has been associated with increased virulence in mice [30,31].

Given the current co-circulation of the 2009 pandemic H1N1 and seasonal H3N2 viruses, co-infection of these viruses in humans may occur [32]. In this study, the polymerase activity of recombinant vRNP complexes that may be created from the reassortment between these two viruses was examined.

## Results

### Polymerase activity of pdmH1N1, H3N2 and WSN H1N1 vRNP complexes

**Figure 1:**
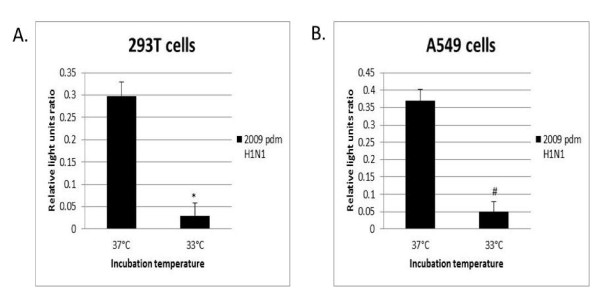
**Polyermase activity of vRNP complexes of 2009 pandemic H1N1 at 33°C and 37°C**. (**a**) 293T cells, 37°C and 33°C. (B) A549 cells, 37°C and 37°C. Polymerase activity as reflected by the normalized relative light units ratio (mean ± standard deviation, n = 3) at 37°C compared to 33°C, * represents statistical significance at *p *< 0.03, and # represents statistical significance at *p *< 0.01.

**Figure 2:**
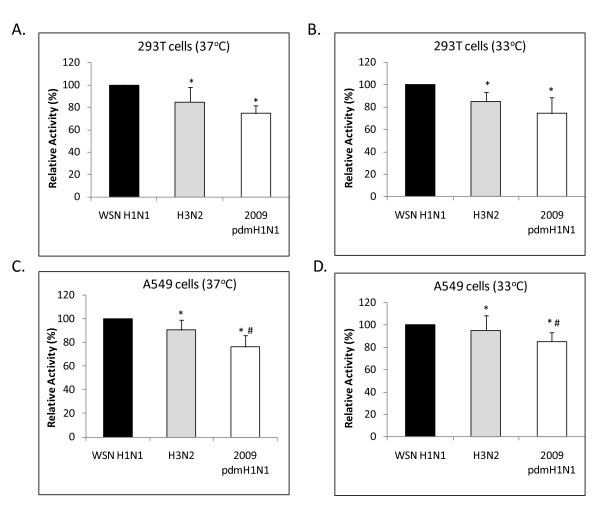
**Polyermase activity of vRNP complexes of 2009 pandemic H1N1, seasonal H3N2 and WSN H1N1**. (**a**) 293T cells, 37°C. (**b**) 293T cells, 33°C. (**c**) A549 cells, 37°C. (**d**) A549 cells, 33°C. Polymerase activity as reflected by the normalized relative light units was expressed as relative activity (mean ± standard deviation, n = 3) compared to the reference strain WSN H1N1, * represents statistical significance at *p *< 0.01 compared to WSN H1N1, and # represents statistical significance at *p *< 0.05 compared to H3N2.

The results of luciferase assays performed with the parental 2009 pdmH1N1, H3N2, and WSN H1N1 vRNPs are shown in Figures 1 and 2. All recombinant vRNPs showed polymerase activity in both A549 and 293T cells under 33°C or 37°C incubation. A significantly lower level of polymerase activity for the 2009 pdmH1N1 vRNP was observed at 33°C compared to 37°C for both cells (293T cells RLU ratio: 0.030 vs 0.298, *P *= 0.03; A549 cells RLU ratio: 0.050 vs 0.371, *P *= 0.01) (Figure 1), whereas no significant differences with respect to incubation temperature were observed for WSN and H3N2 vRNPs (Figure 2).

The polymerase activity of 2009 pdmH1N1 vRNP as recorded from 293T cells incubated at 37°C was significantly lower than that of WSN H1N1 (RLU ratio: 0.498 vs 0.612, *P *= 0.01), and this observation was reproduced in A549 cells (RLU ratio: 0.402 vs 0.533, *P *= 0.01). Furthermore, in A549 cells, the polymerase activity of 2009 pdmH1N1 vRNP was significantly lower than that of H3N2 at 33°C (RLU ratio: 0.358 vs 0.396, *P *= 0.04) and at 37°C (RLU ratio: 0.402 vs 0.479, *P *= 0.01), respectively (Figure 2).

### Polymerase activity of reassortant vRNPs derived from 2009 pdmH1N1 and H3N2

**Figure 3:**
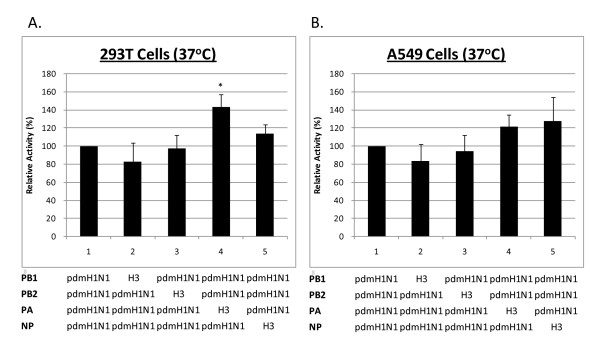
**Polymerase activity of 2009 pdmH1N1 vRNP substituted with H3N2 PB1, PB2, PA and NP**. Recombinant vRNPs were transfected into (**a**) 293T and (**b**) A549 cells at an incubation temperature of 37°C. Polymerase activity as reflected by the normalized relative light units was expressed as relative activity (mean ± standard deviation, n = 3) compared to the parent pdmH1N1 vRNP, * represents statistical significance at *p *< 0.05.

Figure 3 shows that the results of luciferase assays obtained from hybrid vRNPs derived from substituting the 2009 pdmH1N1 vRNP with one H3N2 subunit at a time. It was found that substitution with either H3N2 PB1 or H3N2 PB2 resulted in a slightly decrease in polymerase activity, whereas substitution with either H3N2 PA or H3N2 NP resulted in an increase in polymerase activity. The same trend of change in polymerase activity was observed in both 293T and A549 cells. When subjected to statistical analysis, only the substitution with H3N2 PA showed a significant increase in polymerase activity of the 2009 pdmH1N1 vRNP in 293T cells (RLU ratio: 0.34 vs 0.43, *P *= 0.03).

**Figure 4:**
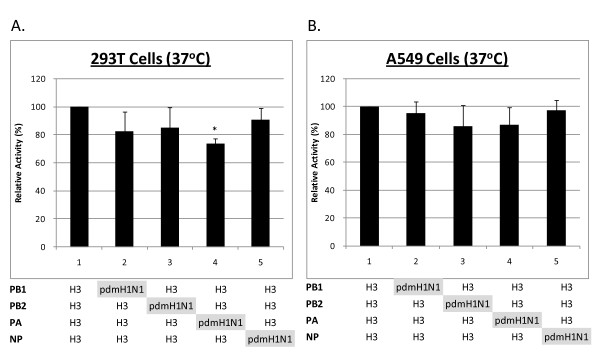
**Polymerase activity of H3N2 vRNPs substituted with 2009 pdmH1N1 PB1, PB2, PA and NP**. Recombinant vRNPs were transfected into (**a**) 293T and (**b**) A549 cells at an incubation temperature of 37°C. Polymerase activity as reflected by the normalized relative light units was expressed as relative activity (mean ± standard deviation, n = 3) compared to the parent H3N2 vRNP, * represents statistical significance at *p *< 0.05.

The results of reciprocal substitution of H3N2 vRNP with 2009 pdmH1N1 subunit are shown in Figure 4. All hybrid vRNPs with either PB1, PB2, PA or NP derived from 2009 pdmH1N1 showed a decrease in polymerase activity. A statistically significant decrease in polymerase activity was observed for the substitution with 2009 pdmH1N1 PA in 293T cells (RLU ratio: 0.57 vs 0.43, *P *= 0.02).

## Discussion

Viral polymerase has a key function in the virus replication cycle and likely to play a role in host adaptation. Previous studies on polymerase activity of influenza were mainly conducted on 293T cells [33]. The results of this study showed that in addition to 293T cells, A549 cells can also serve this purpose. Furthermore, A549 cells could be more appropriate as they are derived from human lung epithelial cells, which is the primary site of replication of influenza viruses.

Our results showed that the polymerase activity of 2009 pdmH1N1 vRNP was significantly lower than WSN H1N1 and H3N2. The difference in activity was more obvious in A549 cells. Although, one could not infer on the transmissibility in humans based on polymerase activity alone, the implication of these *in-vitro *observations deserves further exploration.

It has been reported that avian influenza viruses are adapted for growth in the avian enteric tract with higher temperature (37°C), whereas human influenza viruses are adapted for growth at upper respiratory tract with lower temperature (33°C). It has also been suggested that zoonotic transmission may be limited by temperature differences between the two hosts [3]. In this regard, we compared the polymerase activities of the recombinant vRNPs at 33°C and 37°C. The results showed that the 2009 pdmH1N1 vRNP had a significantly lower activity at 33°C compared to 37°C. It would worthwhile to further investigate whether this was attributed to the avian origin of the PB2 and PA segments of 2009 pdmH1N1 virus.

In addition to the avian-origin PB2 and PA, the vRNP of 2009 pdmH1N1 virus is composed of a human-origin PB1 and a classic swine-origin NP. We hypothesized that substitution of one of these vRNP subunits with a human (H3N2)-origin subunit could confer a change in polymerase activity. The results of our vRNP subunit substitution experiment showed that each of the 2009 pdmH1N1 vRNP subunit could be substituted by a corresponding H3N2 subunit, and the hybrid vRNPs still retained a polymerase activity comparable (~ +/- 20%) to the parent vRNP. Among these substitutions, an H3N2-origin PA conferred a statistically significant increase in the level of polymerase activity in 293T cells. In reciprocal, a hybrid recombinant H3N2 vRNP substituted with 2009 pdmH1N1 PA subunit showed a significant decrease in polymerase activity in 293T cells. The increase in the level of polymerase activity in 293T cells was more significant than that in A549 cells. Since PA forms a dimer with PB1, the increase in activity observed in our study might due to a better compatibility of H3N2 PA with 2009 pdmH1N1 PB1 and vice versa. Our observations are in line with a previous study on H5N1, H1N1 and H3N2 subtype viruses, where PA was found to be a major determining factor responsible for the enhanced polymerase activity of H5N1, while the other subunits had little effect [34,35].

Since the 2009 pdmH1N1 PB2 was originated from an avian subtype lacking the human adaptation mutation E627K [12,36-40], one might expect that the PB2 subunit of H3N2 could increase the polymerase activity of 2009 pdmH1N1 vRNP. As yet, when the 2009 pdmH1N1 vRNP was substituted with a human (H3N2) PB2, a slightly decrease in polymerase active was observed in both 293T and A549 cells. Nevertheless, one should note that the subunits of vRNP are known to interact with each other. For instance, PB2 interacts with PB1 [41-43] and possibly with PA [44]. Substituting the 2009 pdmH1N1 vRNP with a PB2 of H3N2 origin may affect these interactions.

## Conclusions

Overall, our data suggest that hybrid vRNPs resulted from reassortment between 2009 pdmH1N1 and H3N2 viruses could still retain a substantial level of polymerase activity. Substituion of the subunit PA confers the most prominent effect on polymerase activity. Further studies to explore the determinants for polymerase activity of influenza viruses in associate with other factors that limit host specificity are warrant.

## Methods

### In-vitro cell models

Two human cell lines of different tissue origin were used as an *in-vitro *model to examine the polymerase activity of vRNP complexes. The A549 cells were derived from human alveolar basal epithelial adenocarcinoma (ATCC, CCL-185, Rockville, MD, USA), and the 293T cells were derived from human embryonic kidney (ATCC, CRL-11268). These cells were maintained in minimum essential medium (MEM) supplemented with 10% fetal bovine serum (FBS), 1% penicillin, and 1% streptomycin (all from Gibco, Life Technology, Rockville, Md., USA) at 33°C or 37°C in a 5% CO_2 _incubator.

### Virus strains

Three influenza strains were used for preparing cDNA clones correspond to the respective vRNP subunits. The A/Auckland/1/2009 (H1N1) represented the 2009 pandemic H1N1 virus (2009 pdmH1N1), the A/HongKong/CUHK-22910/2004 (H3N2) represented seasonal H3N2 virus, and the A/WSN/1933 (H1N1) (WSN H1N1) was also included as a reference.

### Expression of recombinant vRNPs

The PB1, PB2, PA and NP-expressing plasmids of WSN H1N1 were kindly provided by Prof. George Brownlee [20,34]. The full-length sequences of PB1, PB2, PA and NP of 2009 pdmH1N1 and H3N2 were amplified using Superscript III reverse transcriptase (Invitrogen, Carlsbad, CA) and PCR with Fusion polymerase (Stratagene, La Jolla, CA, USA). PB1, PA and NP PCR products were inserted into pcDNA3A plasmids [33] using KpnI and NotI restriction sites, whereas the HindIII and NotI restriction sites were used for PB2 PCR product. The DNA sequences of the cloned genes were checked by direct sequencing.

The expression plasmids were used to generate recombinant vRNPs as described previously [45]. Briefly, 1 μg of each plasmid was transfected into 293T or A549 cells by Lipofectamine 2000 (Invitrogen) according to the manufacturer's instructions. The medium of transfected cells was replaced by MEM with 10% FBS, 1% penicillin and 1% streptomycin (all from Gibco, Life Technology) at 6 h post-transfection.

### Luciferase reporter assay for viral polymerase activities

A series of different combinations of PB2, PB1, PA and NP protein expression plasmids and the pPolI-NP-Luc were co-transfected into 293T or A549 cells. In addition, a reporter plasmid pGL4.73[hRluc/SV40], encoding a Renilla luciferase gene, was co-transfected to serve as a control for normalizing the transfection efficiency between experiments. At 48 h post-transfection, the polymerase activities of recombinant vRNPs were determined. According to the manufacturer's instructions, cells were lysed by using the Steady-Glo assay reagent (Promega, Madison, WI, USA) for 10 min and the luminescence was measured by a microplate luminometer (Wallac VICTOR3, PerkinElmer, Norwalk, CT).

### Data analysis

All data were generated from three separate experiments. The results of the luciferase reporter assay were recorded as relative light units (RLU). The ratio of RLU normalized with the internal control were used for comparing the polymerase activities between different vRNPs. Differences in normalized RLU ratio between two vRNPs were compared by the Student's*t*-test. *P*-values less than 0.05 were regarded as significant.

## Abbreviations

CDC: Centers for disease control and prevention; cDNA: Complementary deoxyribonucleic acid; cRNA: Complementary ribonucleic acid; D701N: A change of amino acid 701 from aspartic acid to asparagine; E627K: A change of amino acid 627 from glutamic acid to lysine; FBS: Fetal bovine serum; MEM: Minimum essential medium; mRNA: Messenger ribonucleic acid; NP: Nucleoprotein; PA: Polymerase acidic protein; PB1: Polymerase basic protein 1; PB2: Polymerase basic protein 2; RLU: Relative light units; vRNA: Viral ribonucleic acid; vRNP: Viral ribonucleoprotein; WHO: World health organization; 2009 pdmH1N1: 2009 pandemic H1N1 virus; H3N2: A/HongKong/CUHK-22910/2004; WSN H1N1: A/WSN/1933 (H1N1).

## Competing interests

The authors declare that they have no competing interests.

## Authors' contributions

KLKN performed the virus culture, cloning of vRNP plasmids, luciferase reporter assays, recombinant vRNP assays. WYL was responsible for experimental design, analyses and drafting of the manuscript. PKSC was responsible for design and supervision of the study. All authors read and approved the final manuscript.
